# Supplementary material for: Designing and validating a Markov model for hospital-based addiction consult service impact on 12-month drug and non-drug related mortality
Source: PLoS One. 2021 Sep 10;16(9):e0256793. doi: 10.1371/journal.pone.0256793 (PMC8432751; doi:10.1371/journal.pone.0256793)
Supplement: S1 File — (DOCX) [file pone.0256793.s001.docx]

**S1 Table. Model fit statistics**

|  | **Sample Size or Confidence Interval percentage** | **Expected log pointwise predictive density** | **Effective number of parameters** | **Leave-one-out information criterion** | **Pareto k diagnostic values (all at least “ok”)** |
| --- | --- | --- | --- | --- | --- |
| **Referral to ACS** | **0.1% sample size** | -1108.8 | 10.6 | 2217.6 | Yes |
|  | **1% sample size** | -1273.0 | 6.9 | 2546.1 | Yes |
|  | **5% sample size** | -1614.2 | 5.6 | 3228.5 | Yes |
|  | **10% sample size** | -1949.8 | 7.4 | 3899.5 | Yes |
|  | **80% confidence interval** | -1103.8 | 11.2 | 2207.6 | Yes |
|  | **85% confidence interval** | **-1109.9** | **10.7** | **2219.7** | **Yes** |
|  | **90% confidence interval** | -1118.6 | 10.6 | 2237.2 | Yes |
|  | **95% confidence interval** | -1132.2 | 10.4 | 2264.5 | Yes |
| **Engagement in post-discharge OUD treatment** | **0.1% sample size** | **-397.1** | **12.4** | **794.2** | **Yes** |
|  | **1% sample size** | -406.6 | 10 | 813.3 | Yes |
|  | **5% sample size** | -457.8 | 7.4 | 915.7 | Yes |
|  | **10% sample size** | -511.3 | 7.2 | 1022.5 | Yes |
|  | **80% confidence interval** | -415.6 | 9.6 | 831.3 | Yes |
|  | **85% confidence interval** | -419.2 | 9.5 | 838.5 | Yes |
|  | **90% confidence interval** | -424.3 | 8.9 | 848.6 | Yes |
|  | **95% confidence interval** | -434.0 | 8.4 | 868.0 | Yes |
| **Twelve-month drug-related mortality** | **0.1% sample size** | **-95.9** | **8.7** | **191.8** | **Yes** |
|  | **1% sample size** | -98.5 | 6.3 | 197.1 | No |
|  | **5% sample size** | -109.1 | 5 | 218.2 | No |
|  | **10% sample size** | -116.6 | 5.1 | 233.1 | No |
|  | **80% confidence interval** | -104.3 | 6.6 | 208.7 | No |
|  | **85% confidence interval** | -104.9 | 6.3 | 209.8 | No |
|  | **90% confidence interval** | -106.1 | 6.2 | 212.3 | No |
|  | **95% confidence interval** | -107.3 | 5.8 | 214.6 | No |
| **Twelve-month non-drug-related mortality** | **0.1% sample size** | **-189.4** | **14.0** | **378.8** | **Yes** |
|  | **1% sample size** | -189.9 | 11.4 | 379.8 | Yes |
|  | **5% sample size** | -192.8 | 8.7 | 385.7 | Yes |
|  | **10% sample size** | -194.3 | 7.4 | 388.7 | No |
|  | **80% confidence interval** | -195.2 | 7.6 | 390.4 | Yes |
|  | **85% confidence interval** | -195.5 | 7.2 | 391.0 | Yes |
|  | **90% confidence interval** | -196.1 | 7.0 | 392.2 | Yes |
|  | **95% confidence interval** | -196.9 | 6.7 | 393.8 | No |
