# Supplementary material for: Designing and validating a Markov model for hospital-based addiction consult service impact on 12-month drug and non-drug related mortality
Source: PLoS One. 2021 Sep 10;16(9):e0256793. doi: 10.1371/journal.pone.0256793 (PMC8432751; doi:10.1371/journal.pone.0256793)
Supplement: S2 File — (DOCX) [file pone.0256793.s002.docx]

**S2. Estimates from classical and Bayesian logistic regression models, and prior-posterior plots**

|  |  | **Adjusted logistic regression output**  **OR (95% CI)** | **Bayesian logistic regression output**  **OR (95% Posterior Interval)** |
| --- | --- | --- | --- |
| **Referral to ACS** | **Intercept** | 0.03 (0.02, 0.05) | 0.04 (0.02, 0.06) |
|  | **Age** | 0.97 (0.96, 0.98) | 0.97 (0.96, 0.98) |
|  | **Gender (ref= female)** | 1.78 (1.38, 2.31) | 0.94 (0.75, 1.18) |
|  | **Race: unknown** | 1.22 (0.91, 1.61) | 1.17 (0.89, 1.54) |
|  | **Race: Not White** | 0.90 (0.51, 1.48) | 2.04 (1.40, 2.90) |
|  | **Ethnicity: Hispanic** | 0.93 (0.44, 1.74) | 3.54 (2.43, 5.06) |
|  | **Alcohol Use Disorder** | 1.26 (0.67, 2.18) | 1.90 (1.12, 3.09) |
|  | **Stimulant Use Disorder** | 1.68 (1.15, 2.39) | 2.57 (1.88, 3.49) |
|  | **Length of stay** | 1.02 (1.02, 1.03) | 1.02 (1.01, 1.03) |
|  | **Rural residence** | 0.39 (0.26, 0.56) | 0.69 (0.51, 0.92) |
|  | **On medication for OUD at time of hospital admission** | 1.00 (0.71, 1.37) | 1.21 (0.91, 1.61) |
|  | **Previously admitted to the hospital** | 2.19 (1.69, 2.84) | 2.25 (1.77, 2.85) |
|  | **CDPS Score** | 1.24 (1.15, 1.32) | 1.25 (1.17, 1.33) |
| **Engagement in post-discharge OUD treatment** | **Intercept** | 0.10 (0.04, 0.20) | 0.12 (0.05, 0.28) |
|  | **Age** | 1.00 (0.98, 1.01) | 1.00 (0.98, 1.01) |
|  | **Gender (ref= female)** | 0.98 (0.67, 1.44) | 0.93 (0.66, 1.31) |
|  | **Race: unknown** | 1.03 (0.67, 1.59) | 0.99 (0.64, 1.50) |
|  | **Race: Not White** | 1.98 (1.01, 3.83) | 2.08 (1.15, 3.74) |
|  | **Ethnicity: Hispanic** | 0.71 (0.23, 1.96) | 0.69 (0.29, 1.61) |
|  | **Alcohol Use Disorder** | 0.71 (0.27, 1.71) | 1.20 (0.55, 2.55) |
|  | **Stimulant Use Disorder** | 1.13 (0.63, 1.96) | 1.20 (0.71, 2.04) |
|  | **Length of stay** | 1.00 (0.99, 1.01) | 1.00 (0.99, 1.01) |
|  | **Rural residence** | 0.48 (0.28, 0.81) | 0.55 (0.34, 0.88) |
|  | **On medication for OUD at time of hospital admission** | 40.94 (25.55, 67.54) | 31.60 (20.27, 50.10) |
|  | **Previously admitted to the hospital** | 0.91 (0.62, 1.33) | 0.92 (0.64, 1.33) |
|  | **CDPS Score** | 1.00 (0.88, 1.13) | 1.00 (0.89, 1.13) |
|  | **Referred to ACS** | 6.91 (4.56, 10.64) | 6.24 (4.21, 9.32) |
| **Twelve-month drug-related mortality** | **Intercept** | 0.0013 (0.0001, 0.02) | 0.01 (0.001, 0.04) |
|  | **Age** | 1.02 (0.99, 1.06) | 1.02 (0.99, 1.05) |
|  | **Gender (ref= female)** | 1.76 (0.69, 4.49) | 0.62 (0.30, 1.25) |
|  | **Race: unknown** | 2.43 (0.85, 6.93) | 2.26 (0.84, 5.87) |
|  | **Race: Not White** | 2.22 (0.51, 9.64) | 4.28 (1.42, 12.32) |
|  | **Ethnicity: Hispanic** | 0.29 (0.01, 5.86) | 1.60 (0.40, 5.36) |
|  | **Alcohol Use Disorder** | 1.98 (0.54, 7.21) | 2.97 (0.95, 8.29) |
|  | **Stimulant Use Disorder** | 0.56 (0.10, 3.32) | 0.68 (0.14, 2.53) |
|  | **Length of stay** | 1.01 (0.996, 1.03) | 1.00 (0.97, 1.02) |
|  | **Rural residence** | 0.86 (0.26, 2.83) | 1.20 (0.47, 2.91) |
|  | **On medication for OUD at time of hospital admission** | 2.99 (0.82, 10.96) | 1.92 (0.64, 5.50) |
|  | **Previously admitted to the hospital** | 4.41 (1.50, 12.98) | 3.45 (1.48, 8.54) |
|  | **CDPS Score** | 1.09 (0.81, 1.45) | 1.03 (0.78, 1.33) |
|  | **Filled naloxone prescription within 30 days of hospital discharge** | 1.12 (0.05, 23.52) | 3.61 (0.89, 12.96) |
|  | **Engaged in post-discharge OUD treatment** | 0.24 (0.05, 1.11) | 0.39 (0.12, 1.16) |
| **Twelve-month non-drug-related mortality** | **Intercept** | 0.001 (0.0002, 0.006) | 0.002 (0.0003, 0.01) |
|  | **Age** | 1.05 (1.03, 1.07) | 1.05 (1.03, 1.07) |
|  | **Gender (ref= female)** | 1.27 (0.73, 2.23) | 0.99 (0.58, 1.69) |
|  | **Race: unknown** | 1.30 (0.60, 2.84) | 1.19 (0.54, 2.51) |
|  | **Race: Not White** | 0.51 (0.15, 1.78) | 0.85 (0.28, 2.23) |
|  | **Ethnicity: Hispanic** | 2.98 (1.04, 8.57) | 3.43 (1.22, 8.89) |
|  | **Alcohol Use Disorder** | 0.25 (0.04, 1.43) | 0.37 (0.07, 1.40) |
|  | **Stimulant Use Disorder** | 0.46 (0.14, 1.57) | 0.46 (0.12, 1.40) |
|  | **Length of stay** | 1.00 (0.99, 1.01) | 1.00 (0.98, 1.01) |
|  | **Rural residence** | 1.01 (0.52, 1.97) | 1.14 (0.59, 2.12) |
|  | **On medication for OUD at time of hospital admission** | 1.15 (0.41, 3.26) | 1.23 (0.47, 3.10) |
|  | **Previously admitted to the hospital** | 1.47 (0.84, 2.59) | 1.43 (0.82, 2.51) |
|  | **CDPS Score** | 1.57 (1.35, 1.83) | 1.57 (1.35, 1.84) |
|  | **Filled naloxone prescription within 30 days of hospital discharge** | 0.32 (0.02, 6.17) | 0.81 (0.11, 3.86) |
|  | **Engaged in post-discharge OUD treatment** | 0.76 (0.30, 1.92) | 0.68 (0.28, 1.58) |

**S2 Figure 1. Prior-posterior plots for referral to addiction consult service
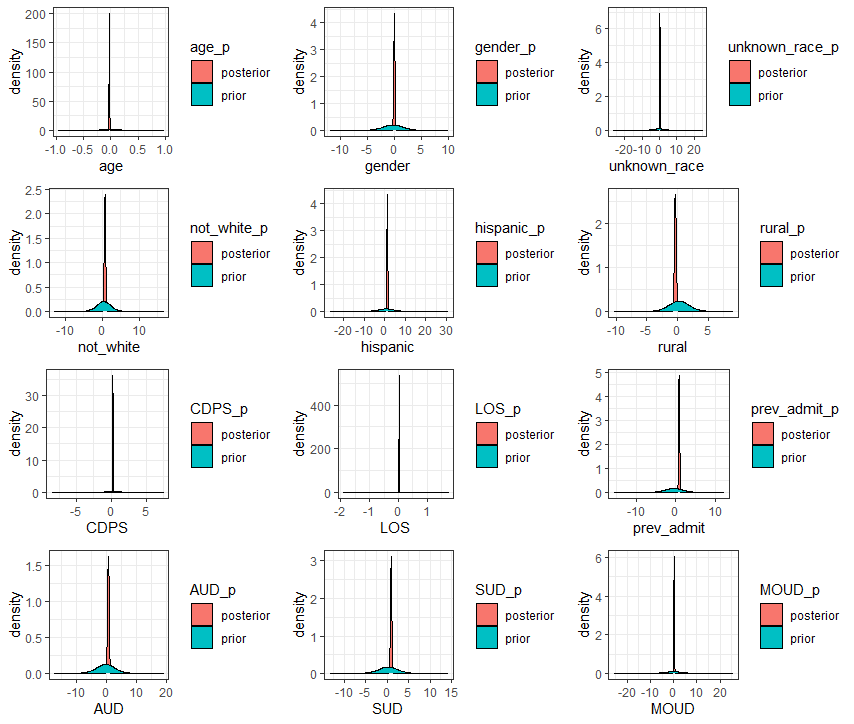
**

**S2 Figure 2. Prior-posterior plots for engagement in post-discharge OUD treatment
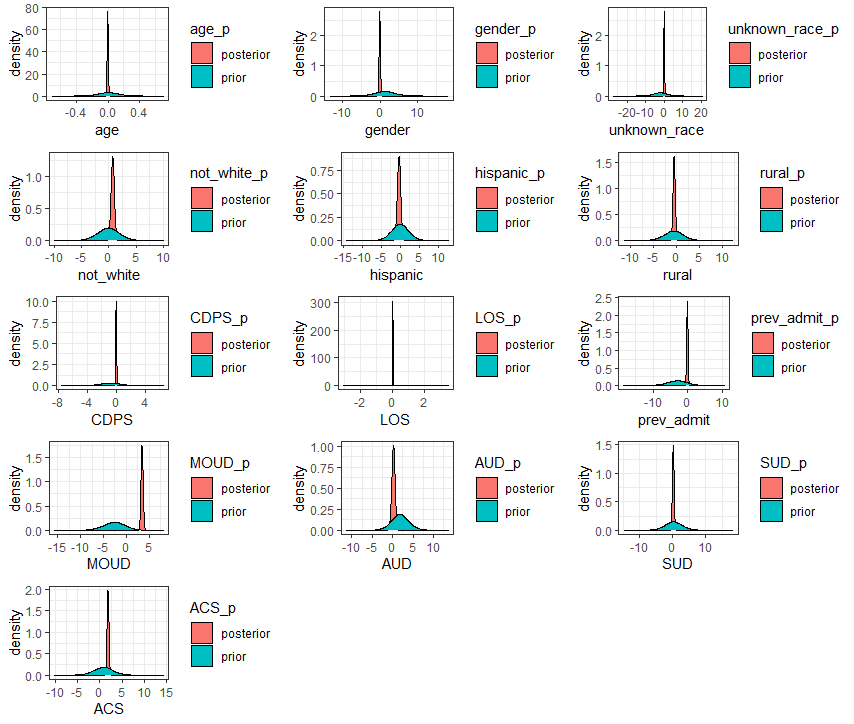
**

**S2 Figure 3. Prior-posterior plots for drug-related mortality at 12 months**

**
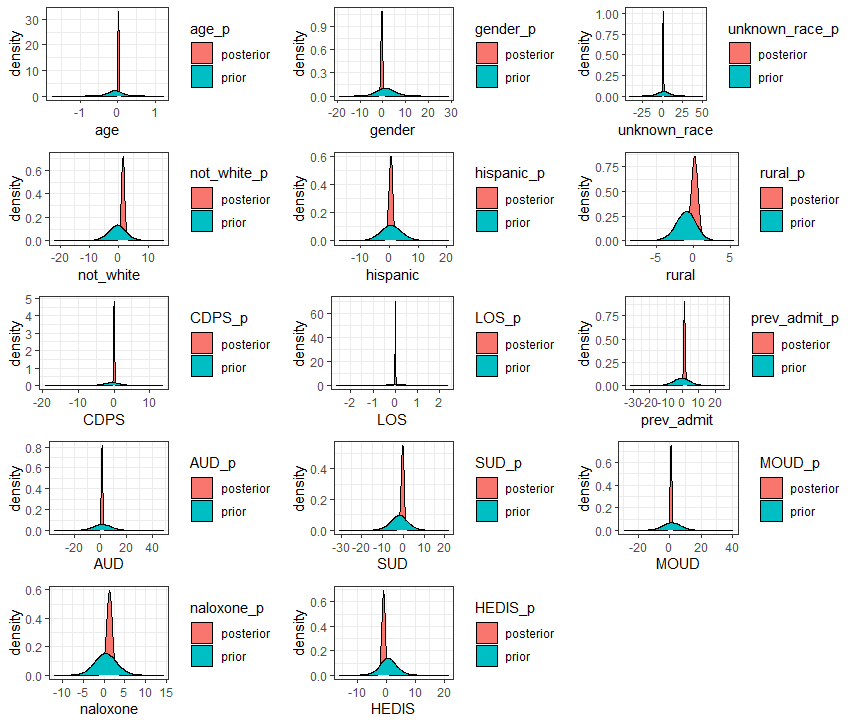
**

**S2 Figure 4. Prior-posterior plots for non-drug related mortality at 12 months**

**
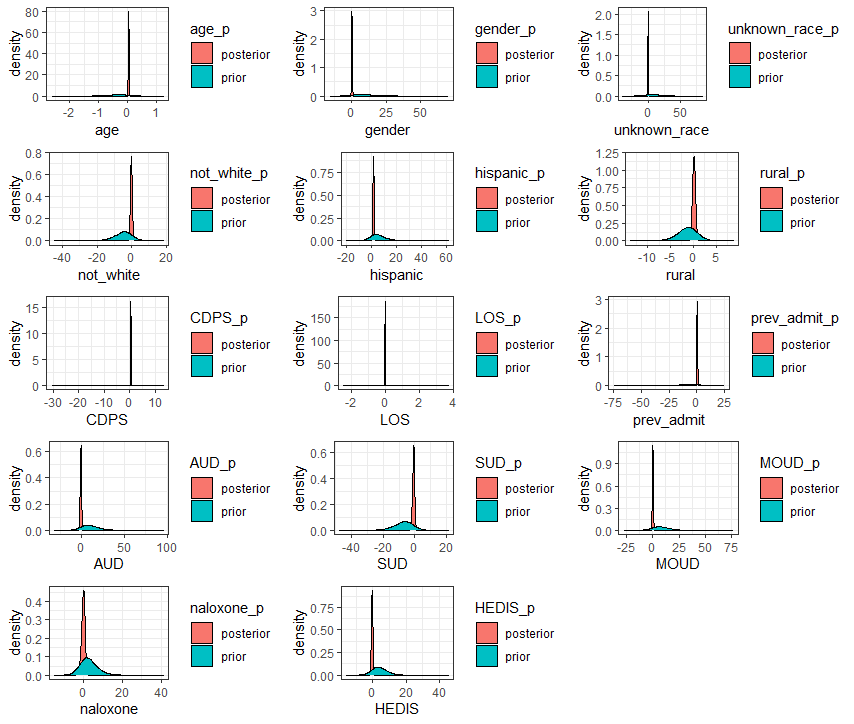
**
