# Supplementary material for: Designing and validating a Markov model for hospital-based addiction consult service impact on 12-month drug and non-drug related mortality
Source: PLoS One. 2021 Sep 10;16(9):e0256793. doi: 10.1371/journal.pone.0256793 (PMC8432751; doi:10.1371/journal.pone.0256793)
Supplement: S3 File — (DOCX) [file pone.0256793.s003.docx]

**S3. Non-technical model description**

**Model and purpose**

The purpose of this model is to understand how people who are hospitalized with opioid use disorder progress through care, from the time they are hospitalized through 12 months after they are discharged. We are especially interested in addiction care in Oregon, but the model could be used in states and settings other than ours.

**Types of applications designed to address**

The model is designed to understand mortality, from drug-related causes like overdose, and from non-drug related causes like heart attack, in the twelve-months after discharge from the hospital. We wanted to know how referral to addiction consult services, a specialized team in the hospital that cares for patients admitted with addiction, impact post-discharge engagement in treatment for opioid use disorder and death within twelve months of discharge.

**Sources of funding and their role**

This work was funded by the National Institutes of Health. The funder of the study had no role in study design, data collection, data analysis, data interpretation, or writing of the report. Dr. Korthuis serves as principal investigator for NIH-funded studies that accept donated study medication from Alkermes (extended-release naltrexone) and Indivior (buprenorphine).

**Structure**

Here is our model structure (Fig 1). Once patients were admitted to a hospital in Oregon from 2015 to 2018 and diagnosed with opioid use disorder, they could be referred to see an addiction consult service, or not. After they were discharged, they could engage in post-discharge care for opioid use disorder, or not. At twelve months, we looked to see if they were still alive, or if they had died, if it was from a drug-related, or non-drug related cause. We used Oregon Medicaid claims data to gather information. We also used information from experts in addiction. We combined the Medicaid data with the expert information using a technique called Bayesian analysis.

**
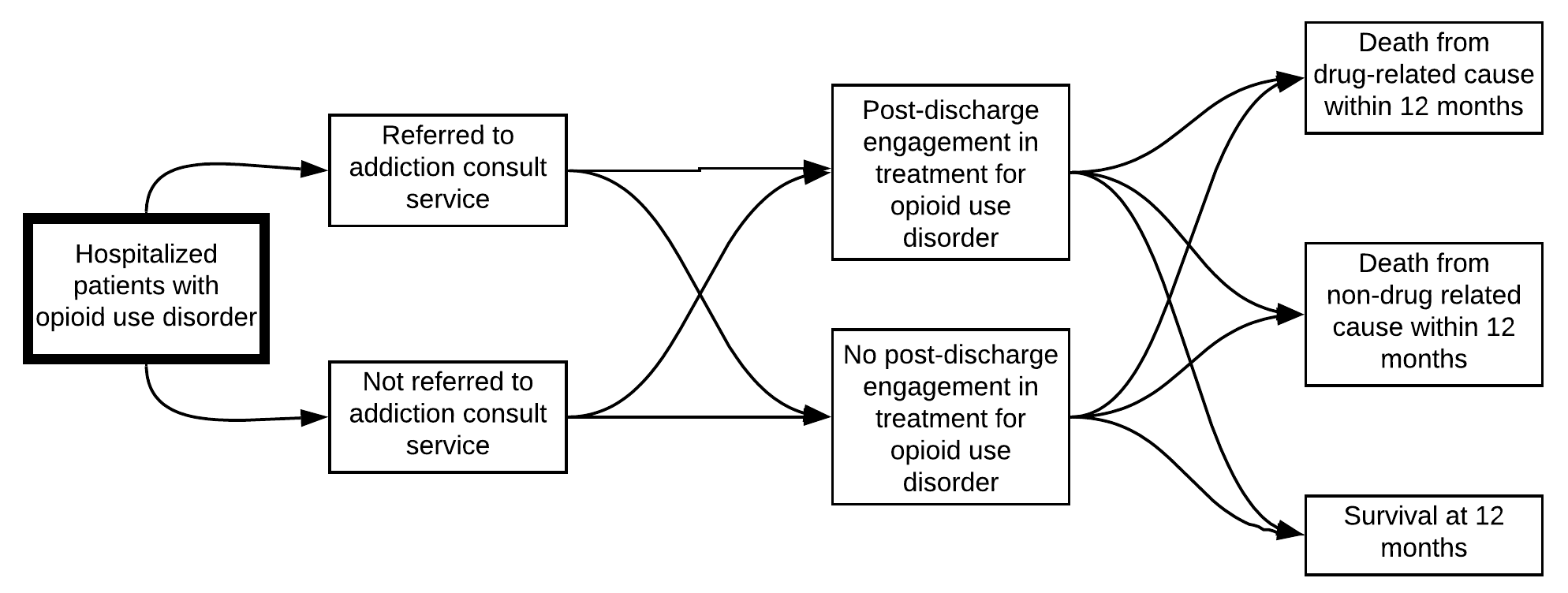
**

**Model validation and summary of results**

We validated our model a few ways. First, we spoke to experts about what we had planned, and they agreed that this model represented how patients move through care in real life, and that the questions we had about survival were important to answer. Next, once we fit our model, we checked to make sure our actual number of deaths matched the number modeled. Then, we compared our model estimates to another model, built independently by a research team in Boston, to compare results. After that, we used high-quality data from published studies to see if our model could accurately predict what happened to patients in published studies. We found that, in general, our model better matched observed estimates from Oregon than the national model, which suggests that using our model with local data in different contexts may provide more accurate information in those settings.

**Main limitations for its intended applications**

The main limitation of this model is that it does not include non-addiction consult service addiction care in hospitals. We are not able to tell how much of a difference there might be from addiction consult services versus standard addiction care provided by other types of doctors. However, we know that there are additional benefits from addiction consult services: they can help transform hospital environments more broadly to better care for patients with addiction. Additionally, very few people receive addiction care while in the hospital in general.

**Reference to the model’s technical documentation**

For more information, see our paper (cite).
