# Supplementary material for: Designing and validating a Markov model for hospital-based addiction consult service impact on 12-month drug and non-drug related mortality
Source: PLoS One. 2021 Sep 10;16(9):e0256793. doi: 10.1371/journal.pone.0256793 (PMC8432751; doi:10.1371/journal.pone.0256793)
Supplement: Attachment — Submitted filename: review_OUD.pdf [file pone.0256793.s004.pdf]

Review of “Designing and validating a Markov model for hospital-based addiction consult service impact on 12-month drug and non-drug related mortality”

The paper presents a Markov-chain based model to analyze data from patients suffering from OUD. A Bayesian regression-based model is used to estimate the transition probabilities of different stages. The data set is quite large, and the work is described well. While I do believe this work will benefit decision makers, the authors definitely need to do some additional analysis of what the impact is of the consultation service. What is the difference in mortality when they take the consultation service and/ or do not use the post-discharge treatment? Without this analysis, it is unclear what gap in the literature is filled and what the motivation is for this research: it just provides some data, but not clear how it may be used by decision-makers in public policy.

It is possible to use steady-state probabilities to determine the long-run survival and mortality rates (see e.g., Gosavi et al., 2020). Further, even some basic analysis is possible from the probabilities in Fig 2: what is the probability that someone who took advantage of the consulting service survived, vs that of someone who did not?

Typo: In Discussion: “Second, claims data is often inaccurate in classifying
patient race and ethnicity” → Do you mean “…claims *that* data…? There is a grammar error here. Please fix. Also, please note that data *are* and not *is*.

Gosavi, A, Murray, S.L., and Karagiannis, N. (2020). A Markov Chain Approach for Forecasting Progression of Opioid Addiction. *Proceedings of the Industrial and Systems Engineering Annual Conference*, Virtual, L. Cromarty, R. Shirwaiker, P. Wang, eds.
